# DTPA-Bound Planar Catechin with Potent Antioxidant Activity Triggered by Fe^3+^ Coordination

**DOI:** 10.3390/antiox12020225

**Published:** 2023-01-18

**Authors:** Kiyoshi Fukuhara, Ikuo Nakanishi, Kohei Imai, Mirei Mizuno, Ken-ichiro Matsumoto, Akiko Ohno

**Affiliations:** 1 Division of Organic and Medicinal Chemistry, School of Pharmacy, Showa University, Shinagawa-ku, Tokyo 142-8555, Japan; 2 Quantum RedOx Chemistry Team, Institute for Quantum Life Science (iQLS), Quantum Life and Medical Science Directorate, National Institutes for Quantum Science and Technology (QST), Inage-ku, Chiba 263-8555, Japan; 3 Quantitative RedOx Sensing Group, Department of Radiation Regulatory Science Research, National Institute of Radiological Sciences (NIRS), Quantum Life and Medical Science Directorate, National Institutes for Quantum Science and Technology (QST), Inage-ku, Chiba 263-8555, Japan; 4 Division of Risk Assessment, Center for Biological Safety & Research, National Institute of Health Sciences, Kawasaki-ku, Kawasaki, Kanagawa, Yokohama 210-9501, Japan

**Keywords:** catechin, diethylenetriaminepentaacetic acid, Fe^3+^, antioxidant activity

## Abstract

In diseases related to oxidative stress, accumulation of metal ions at the site of pathogenesis results in the generation of reactive oxygen species (ROS) through the reductive activation of oxygen molecules catalyzed by the metal ions. If these metals can be removed and the generated ROS can be strongly scavenged, such diseases can be prevented and treated. Planar catechins exhibit stronger radical scavenging activity than natural catechins and can efficiently scavenge hydroxyl radicals generated by the Fenton reaction without showing pro-oxidant effects, even in the presence of iron ions. Hence, in the current study, we designed a compound in which diethylenetriaminepentaacetic acid (DTPA), a metal chelator, was bound to a planar catechin with enhanced radical scavenging activity by immobilizing the steric structure of a natural catechin to be planar. This compound showed almost no radical scavenging activity due to intramolecular hydrogen bonding of DTPA with the planar catechins; however, when coordinated with Fe^3+^, it showed more potent radical scavenging activity than planar catechins. Owing to its potent antioxidant activity triggered by metal coordination and its inhibition of ROS generation by trapping metal ions, this compound might exert excellent preventive and therapeutic effects against oxidative stress-related diseases.

## 1. Introduction

Excessive production of reactive oxygen species (ROS) in living cells causes damage to proteins, nucleic acids, lipids, and other substances in living organisms, leading to their dysfunction. Oxidative stress caused by ROS generation is involved in the pathogenesis of various human diseases, such as neurodegenerative diseases, metabolic diseases, and cancer [1,2,3,4]. The most basic symptomatic treatment for preventing the onset and progression of diseases involving oxidative stress is to prevent and reduce ROS toxicity [5]. There are two methods for preventing ROS toxicity: suppressing ROS generation by controlling its production and scavenging the generated ROS.

Vitamins C and E and many natural polyphenols inhibit ROS toxicity by directly scavenging ROS generated during physiological and pathological processes [6,7]. In Parkinson’s disease, ROS generation induced by mitochondrial dysfunction and autoxidation during catecholamine metabolism is implicated in the pathophysiological mechanisms underlying the selective loss of dopaminergic neurons [8,9]. In Alzheimer’s disease, amyloid β catalyzes the generation of reactive oxygen when it binds with redox-active metal ions with redox activity, such as copper, in the aggregation process, resulting in neurodegeneration that leads to cognitive dysfunction [10,11]. Thus, the intake of foods rich in antioxidants is effective in preventing the onset and progression of Parkinson’s disease and Alzheimer’s disease, in which oxidative stress caused by ROS is involved in the onset and progression of the pathological conditions [12,13,14]. For example, vitamins C and E, resveratrol, and catechins directly scavenge ROS generated in the body and radicals generated during lipid peroxidation reactions triggered by ROS [6,15]. Electrophiles, such as sulforaphane, also scavenge ROS by inducing antioxidant enzymes through Nrf2–Keap1 activation [16]. In addition, natural polyphenols with catechol structures, such as catechins, are oxidized in the body to possess quinone structures, exhibiting similar activating effects on Nrf2–Keap1 [17,18]. Thus, many antioxidants have been shown to exert preventive effects against oxidative stress-related diseases, such as Parkinson’s disease and Alzheimer’s disease, by directly scavenging the generated ROS or inducing antioxidant enzymes [19].

However, iron may be important in catalyzing excessive production of ROS via the Fenton reaction, damaging the cell, particularly when excess iron is available or if iron is not regulated properly [20]. Moreover, it has been reported that abnormal iron accumulation in the brains of patients with Alzheimer’s disease, Parkinson’s disease, and Huntington’s disease causes oxidative stress, leading to cranial nerve damage [21,22]. Therefore, besides scavenging ROS by antioxidants or electrophiles, if the generation of ROS can be suppressed by removing excess iron at the onset site of oxidative stress, it can greatly contribute to the prevention of ROS-related disease onset and progression [23,24].

The iron chelator deferoxamine has recently received attention as a potential therapeutic agent for diseases such as Parkinson’s and Alzheimer’s, in which iron accumulation in the brain is believed to be pathogenic [25]. Furthermore, in patients with viral cirrhosis, hepatic iron overload may cause the generation of free radicals, inducing inflammation and facilitating the development of hepatocellular carcinoma [26,27]. Therefore, in patients with hepatitis C, progression of hepatocellular carcinoma can be prevented by excluding iron to suppress the generation of ROS through an iron-restricted diet [28], phlebotomy [29], and administration of lactoferrin [30], which exhibits iron-chelating properties. However, although either direct scavenging of ROS or suppressing ROS production by removing metals is effective in suppressing oxidative stress, sufficient preventive and therapeutic effects on oxidative stress-related diseases have not been observed.

**Figure 1:**
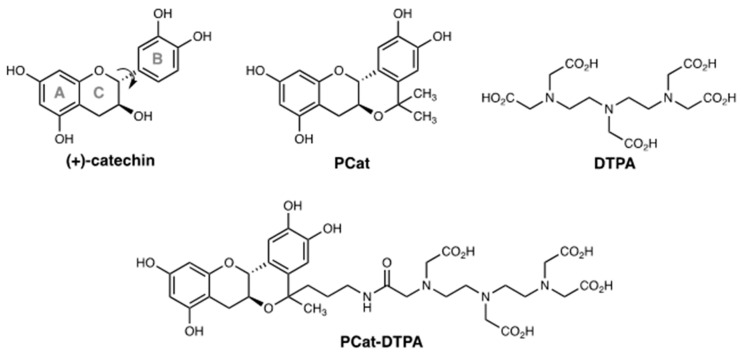
Structures of (+)-catechin, PCat, DTPA, and PCat-DTPA.

Therefore, to effectively suppress disease-related ROS toxicity, we considered that antioxidants must act as inhibitors of the ROS formation process and scavengers of the generated ROS. We previously synthesized a planar catechin analog in which the catechol (B-ring) and chroman moieties (AC-ring) within the (+)-catechin structure were constrained to be planar (Figure 1) [31]. Planar catechins have stronger radical scavenging activity than natural catechins and can efficiently scavenge hydroxyl radicals generated by the Fenton reaction without showing pro-oxidant effects, even in the presence of iron ions [32]. In this study, we aimed to design a dual-functional antioxidant (PCat-DTPA) in which a planar catechin was conjugated to DTPA as a metal chelator. This compound is expected to exhibit excellent antioxidant activity, as DTPA could suppress the formation of ROS by removing iron ions from the ROS formation system through its coordination and PCat could scavenge the generated ROS by its strong radical scavenging activity. The findings of this study might be valuable in the development of lead compounds effective against oxidative stress-related diseases.

## 2. Materials and Methods

### 2.1. General Methods

The reagents and solvents were of commercial origin (Wako Chemicals, Tokyo, Japan; Tokyo Chemical Industry, Tokyo, Japan; Sigma-Aldrich, St. Louis, MO, USA) and were used without further purification. (+)-Catechin (Sigma-Aldrich) was dried under vacuum for 12 h. All reactions were monitored by thin-layer chromatography on silica gel 60 F254 (0.25 nm, Merck KGaA, Darmstad, Germany) using UV light and a sulfuric acid–phosphomolybdic acid solution in ethanol for detection. Column chromatography was performed using silica gel 60 (0.063–0.200 mm, Merck) unless otherwise stated. ^1^H and ^13^C NMR spectra were recorded with a JEOL JNMAL-400 (400 MHz) NMR spectrometer using CDCl_3_, DMSO-*d*_6_, or CD_3_OD as the solvent and tetramethylsilane as the internal reference. Mass spectra were measured using a JMS-700V (JEOL) mass spectrometer. The purity of all compounds was determined to be >95% using ^1^H and ^13^C NMR spectroscopy.

### 2.2. Synthesis of 2-PCat-DTPA Compounds

#### 2.2.1. Ethyl 3-((6a*S*,12a*R*)-2,3,8,10-tetrahydroxy-5-methyl-5,6a,7,12a-tetrahydroisochromeno [4,3-*b*]chromen-5-yl)propanoate (**1**)

Trimethylsilyl trifluoromethanesulfonate (TMSOTf, 2.17 mL, 12 mmol) was slowly added to a solution of (+)-catechin (2.90 g, 10 mmol) and ethyl 4-oxovalerate (5.71 mL 40 mmol) in dry tetrahydrofuran (THF) (150 mL) at −10 °C. After stirring for 3 h, the mixture was poured into water and extracted using diethyl ether (3 × 150 mL). The organic layer was washed with brine, dried over Na_2_SO_4_, and filtered. The solvent was evaporated, and the residue was purified using flash chromatography (SiO_2_, 10:1 dichloromethane/methanol) to give 3.18 g (76.3%) of 1 as a white powder: m.p. 171–173 °C; ^1^H-NMR (400 MHz, DMSO-d_6_) δ 9.30 (s, 1H), 9.11 (s, 1H), 9.06 (s, 1H), 8.86 (s, 1H), 6.93 (s, 1H), 6.50 (s, 1H), 5.94 (d, *J* = 2.0 Hz, 1H), 5.78 (d, *J* = 2.0 Hz, 1H), 4.38 (d, *J* = 9.2 Hz, 1H), 3.95 (q, *J* = 6.8 Hz, 2H), 3.72 (m, 1H), 2.78 (dd, *J* = 5.6 and 15.2 Hz, 1H), 2.28 (dd, *J* = 10.4 and 15.2 Hz, 1H), 2.01 (t, *J* = 7.6 Hz, 2H), 1.83 (t, *J* = 7.6 Hz, 2H), 1.45 (s, 3H), 1.12 (t, *J* = 7.2 Hz 3H); ^13^C NMR (100 MHz, DMSO-*d*_6_) δ 173.0, 156.7, 156.8, 155.2, 145.1, 144.4, 131.3, 124.7, 111.9, 111.4, 99.1, 95.6, 94.2, 76.1, 72.3, 65.9, 59.7, 28.7, 27.5, 27.2, 26.6, 14.1; HRMS (ESI+) *m*/*z* [M^+^] calcd for C_22_H_24_O_8_ 416.1471, found 416.1473.

#### 2.2.2. Ethyl 3-((6a*S*,12a*R*)-2,3,8,10-tetrakis(benzyloxy)-5-methyl-5,6a,7,12a-tetrahydroisochromeno[4,3-*b*]chromen-5-yl)propanoate (**2**)

K_2_CO_3_ (4.84 g, 35 mmol) was added to a solution of 1 (2.91 g, 7.0 mmol) and benzyl bromide (4.28 mL, 35 mmol) in dry N,N-dimethylformamide (DMF, 50 mL). After stirring overnight at room temperature, the K_2_CO_3_ was filtered. The filtrate was diluted with ethyl acetate, washed with brine, dried over Na_2_SO_4_, and filtered. The solvent was evaporated, and the residue was purified using flash chromatography (SiO_2_, 50:50:1 dichloromethane/n-hexane/methanol) to give 4.95 g (91%) of 2 as a white powder: m.p. 158–160 °C; ^1^H-NMR (400 MHz, DMSO-*d*_6_) δ 7.52-7.15 (m, 20H), 7.07 (s, 1H), 6.57 (s, 1H), 6.42 (d, *J* = 2.0 Hz, 1H), 6.29 (d, *J* = 2.2 Hz, 1H), 5.14 (br s, 4H), 5.07 (br s, 4H), 4.55 (d, *J* = 8.8 Hz 1H), 4.04 (q, *J* = 7.2 Hz, 2H), 3.88 (m, 1H), 3.13 (m, 1H), 2.59 (m, 1H), 2.11 (m, 2H), 1.82 (m, 2H), 1.49 (s, 3H), 1.18 (t, *J* = 7.2 Hz, 3H); ^13^C NMR (100 MHz, DMSO-*d*_6_) δ 173.4, 158.3, 157.4, 152.6, 147.8, 147.3, 137.3, 137.2, 137.1, 132.7, 132.6, 128.5, 128.4, 128.1, 127.9, 127.8, 127.7, 127.6, 127.5, 127.4, 127.3, 126.4, 126.3, 111.8, 110.3, 102.4, 94.8, 93.9, 77.3, 72.3, 70.3, 70.2, 69.9, 69.3, 65.7, 65.5, 28.8, 27.2, 27.0, 26.6, 14.1; HRMS (ESI+) *m*/*z* [M^+^] calcd for C_50_H_48_O_8_ 776.3349, found 776.3352.

#### 2.2.3. 3-((6a*S*,12a*R*)-2,3,8,10-tetrakis(benzyloxy)-5-methyl-5,6a,7,12a-tetrahydroisochromeno[4,3-*b*]chromen-5-yl)propan-1-ol (3)

A solution of **2** (3.89 g, 5.0 mmol) in dry THF (50 mL) was added dropwise to a solution of 1M lithium aluminum hydride (LiAlH_4_) in THF (7.5 mL, 7.5 mmol) and dry THF (50 mL) at 0 °C and then stirred for 10 h at room temperature. The mixture was cooled on ice, and saturated potassium sodium tartrate was added dropwise; subsequently, the mixture was filtered through a small pad of Celite and thoroughly washed with THF. The organic solution was washed with brine, dried over Na_2_SO_4_, and filtered. The solvent was evaporated, and the residue was purified using flash chromatography (SiO_2_, 20:20:1 dichloromethane/n-hexane/methanol) to give 3.27 g (89%) of 3 as a white powder: m.p. 199–203 °C; ^1^H-NMR (400 MHz, DMSO-*d*_6_) δ 7.62–7.15 (m, 20H), 7.07 (s, 1H), 6.55 (s, 1H), 6.42 (d, *J* = 2.0 Hz, 1H), 6.29 (d, *J* = 2.0 Hz, 1H), 5.13 (br s, 4H), 5.08 (br s, 4H), 4.56 (d, *J* = 8.8 Hz, 1H), 3.91 (m, 1H), 3.46 (m, 2H), 3.17 (m, 1H), 2.65 (m, 1H), 1.82 (m, 2H), 1.49 (s, 3H), 1.11 (m, 2H); ^13^C NMR (100 MHz, DMSO-*d*_6_) δ 158.3, 157.4, 155.0, 147.9, 147.8, 137.4, 137.3, 137.2, 137.1, 134.0, 128.5(2), 128.4, 128.1, 127.9, 127.8, 127.7, 127.6, 127.5, 127.4, 127.3, 126.1, 124.6, 111.8, 110.4, 94.8, 93.9, 77.1, 72.7, 70.4, 70.3, 69.4, 69.3, 65.5, 61.1, 39.1, 27.2(2), 27.0; HRMS (ESI+) *m*/*z* [M^+^] calcd for C_48_H_46_O_7_ 734.3244, found 734.3251.

#### 2.2.4. *tert*-Butyl (*tert*-butoxycarbonyl)(3-((6a*S*,12a*R*)-2,3,8,10-tetrakis(benzyloxy)-5-methyl-5,6a,7,12a-tetrahydroisochromeno[4,3-*b*]chromen-5-yl)propyl)carbamate (4)

To a stirred solution of **3** (2.93 g, 4.0 mmol), di-*tert*-butyliminodicarboxylate (3.47 g, 16.0 mmol) and triphenyl phosphate (Ph_3_P, 4.19 g, 16.0 mmol) in 40 mL of dry tetrahydrofuran were added 40% diethyl azodicarboxylate (DEAD) in toluene (7.27 mL, 16.0 mmol) dropwise under argon at 0 °C. The resulting mixture was stirred for 4 h at room temperature before removing the solvent. The residue was purified by flash chromatography (SiO_2_, 1:4 ethyl acetate (EtOAc)/n-hexane) to give 4 (2.17 g, 58%) as a white solid: m.p. 63–66 °C; ^1^H NMR (400 MHz, CDCl_3_) δ 7.55-7.52 (m, 20H), 7.19 (s, 1H), 6.55 (s, 1H), 6.28 (d, *J* = 2.0 Hz, 1H), 6.26 (d, *J* = 2.0 Hz, 1H), 5.24-4.96 (m, 8H), 4.54 (d, *J* = 8.8 Hz, 1H), 3.85 (m, 1H), 3.46 (m, 2H), 3.13 (m, 1H), 2.60 (m, 1H), 1.75 (m, 2H), 1.68 (m, 2H), 1.48 (s, 3H), 1.43 (s, 18H); ^13^C NMR (100 MHz, CDCl_3_) δ 159.0, 158.1, 156.1, 152.8, 148.6, 148.5, 137.4, 137.3, 137.1, 132.4, 128.9, 128.7, 128.3, 128.1, 127.8, 127.7, 127.5, 127.4, 122.6, 112.6, 103.3, 94.8, 93.9, 82.2, 78.1, 73.3, 71.9, 71.5, 71.2, 70.4, 66.4, 46.6, 41.2, 29.9, 28.3, 27.7, 27.3; HRMS (ESI+) *m*/*z* [M + Na^+^] calcd for C_58_H_63_NNaO_10_ 956.4350, found 956.4354.

#### 2.2.5. 3-((6a*S*,12a*R*)-2,3,8,10-tetrakis(benzyloxy)-5-methyl-5,6a,7,12a-tetrahydroisochromeno[4,3-*b*]chromen-5-yl)propan-1-amine (5)

Compound **7** (1.87 g, 2.0 mmol) was dissolved in trifluoroacetic acid (10 mL) and cooled to 0 °C under argon. Next, the reaction mixture was warmed to room temperature and stirred for 3 h. The solvent was removed under reduced pressure; the residue was dissolved in ethyl acetate and washed with a saturated bicarbonate solution. The organic layer was dried over anhydrous sodium sulfate and filtered; subsequently, the solvent was removed. The residue was purified using flash chromatography (SiO_2_, 10:10:1 EtOAc/n-hexane/0.2 M ammonia in methanol) to give 5 (0.675 g, 46%) as a white solid: m.p. 256–259 °C dec; ^1^H NMR (400 MHz, CDCl_3_) δ 7.55–7.51 (m, 20H), 7.05 (s, 1H), 6.54 (s, 1H), 6.29 (d, *J* = 2.0 Hz, 1H), 6.24 (d, *J* = 2.0 Hz, 1H), 5.26-4.97 (m, 8H), 4.49 (d, *J* = 9.2 Hz, 1H), 3.88 (m, 1H), 3.14 (m, 1H), 2.64 (m, 2H), 2.50 (m, 1H), 1.74 (m, 2H), 1.60 (s, 3H), 1.44 (m, 2H); ^13^C NMR (100 MHz, CDCl_3_) δ 159.3, 158.4, 155.9, 147.9, 148.8, 137.6, 137.5, 137.4, 129.4, 129.3, 129.1, 129.0, 128.5, 128.4, 128.2, 128.1, 127.8, 125.9, 113.4, 103.5, 95.2, 94.2, 78.5, 73.6, 72.3, 71.8, 70.7, 70.5, 66.8, 42.5, 41.5, 30.2, 28.3, 27.6; HRMS (ESI+) *m*/*z* [M + H^+^] calcd for C_48_H_48_NO_6_ 734.3482, found 734.3495.

#### 2.2.6. (6a*S*,12a*R*)-5-(5-oxo-7,10,13,13-tetrakis(carboxymethyl)-4,7,10,13-tetra-azatridecane)-5-methyl-5,6a,7,12a-tetrahydroisochromeno[4,3-*b*]chromene-2,3,8,10-tetraol (PCat-DTPA)

To a stirred suspension of compound **8** (184 mg, 0.25 mmol) and diethylenetriaminepentaacetic acid (393 mg, 1.0 mmol) in DMSO (5 mL), 4-(4,6-dimethoxy-1,3,5-triazin-2-yl)-4-methyl-morpholinium chloride (DMT-MM, 69 mg, 0.25 mmol) was added at room temperature. After stirring for 20 h at room temperature, the solvent was removed, and the product was dried under reduced pressure. The residue was dissolved in methanol, suspended in 20% Pd(OH)_2_, and stirred for 10 h under a hydrogen atmosphere. The suspension was then filtered through a small Celite pad. The eluent was concentrated, and the residue was purified using chromatography (Sephadex LH-20, methanol) to give PCat-DTPA (120 mg, 64.1%) as a white solid: ^1^H NMR (400 MHz, CD_3_OD) δ 6.92 (s, 1H), 6.74 (s, 1H), 5.85 (d, *J* = 2.0 Hz, 1H), 5.82 (d, *J* = 2.0 Hz, 1H), 4.32 (d, *J* = 8.8 Hz, 1H), 3.67 (s, 2H), 3.61 (m, 1H), 3.58 (s, 2H), 3.53 (s, 4H), 3.43 (s, 2H), 3.18 (m, 4H), 3.07 (m, 4H), 3.03 (m, 2H), 2.82 (m, 1H), 2.34 (m, 1H), 1.78 (m, 2H), 1.68 (s, 3H), 1.49 (m, 2H); ^13^C NMR (100 MHz, CD_3_OD) δ 171.9, 171.1, 166.8, 156.4, 155.9, 155.1, 144.4, 143.3, 129.6, 128.9, 115.0, 113.3, 100.3, 96.5, 95.3, 76.3, 68.8, 66.8, 59.5, 58.4, 56.3, 54.3, 53.5, 53.2, 51.6, 51.4, 39.9, 36.5, 30.5, 28.5, 24.6; HRMS (ESI−) *m*/*z* [M − H^−^] calcd for C_34_H_43_NO_15_ 747.2725, found 747.2730.

### 2.3. 2,2-Diphenyl-1-picrylhydrazyl (DPPH^•^)-Scavenging Assay

The rates of DPPH^•^-scavenging reactions using test samples in methanol (MeOH) were determined by monitoring the absorbance change at 516 nm due to DPPH^•^ (ε= 1.13 × 10^4^ M^−1^ cm^−1^) after mixing it in MeOH at a volumetric rate of 1:1 using a stopped-flow technique on a UNISOKU REP-1000-02 NM spectrophotometer (Unisoku, Osaka, Japan) at 298 K. The pseudo-first-order rate constants (*k*_obs_) were determined by least-squares curve fitting using a MacBook Pro. The first-order plots of ln(*A* − *A*_∞_) vs. time (*A* and *A*_∞_ were denoted as the absorbance at the reaction time and the final absorbance, respectively) were linear until three or more half-lives with a correlation coefficient ρ > 0.999.

### 2.4. Hydroxyl Radical Scavenging Assay

The hydroxyl radical scavenging activity was measured using the 5,5-dimethyl-1-pyrrolidine-N-oxide (DMPO) spin adduct generated in an iron-containing xanthine oxidase (XO)/hypoxanthine (HX) (Fe-XO/HX) system using the spin-trapping method. The sampling procedure is as follows. All reagents were dissolved in 20mM Tris–HCl buffer solution (pH 7.4). Approximately 50 μL of 1.2 mM HX, 50 μL of 1.2 mM EDTA, 50 μL of 0.06 mM FeCl_3_, and 50 μL of 0.9 M KCl were well mixed in a test tube; subsequently, 3 μL of DMPO and 50 μL of test sample were added. After 50 μL of 0.30 U/mL XO was added to initiate the reaction, a 200 μL aliquot of the mixture was immediately placed into a flat quartz cell to scan and record the ESR spectra. ESR observations were performed using a JES-RE1X spectrometer (JEOL, Tokyo, Japan) using the following settings: modulation width, 0.2 mT; time constant, 0.03 s; and microwave frequency, 9.405 GHz.

### 2.5. Inhibition Assay of DNA Strand Breaks

The effects of antioxidants on DNA strand breakage by the Fenton reaction were measured by converting supercoiled pBR322 plasmid DNA (Form I) into open circular and linear forms (Form II and III). Reactions were carried out in 20 µL (total volume) of 50 mM sodium cacodylate buffer (2.5% DMF, pH 7.2) containing 0.1 µg pBR322 DNA, 10 µM FeCl_2_, 10 µM EDTA, 10 mM H_2_O_2_, and 100 µM of the test samples. After preparing the solution of pBRDNA, FeCl_2,_ and EDTA, the test sample was added, and the reaction was initiated by adding H_2_O_2_. After incubation at 37 °C for 0.5 h, the reaction mixture was treated with 5 µL of loading buffer (100 mM TBE buffer, pH 8.3, containing 30% glycerol, 0.1% bromophenol blue) and applied to a 1% agarose gel. Horizontal gel electrophoresis was performed using 50 mM TBE buffer (pH 8.3). The gels were stained with ethidium bromide (1 µg/mL) for 30 min, destained in water for 30 min, and photographed by UV transillumination.

### 2.6. Calculation Methods

The geometries and orbital compositions of the highest occupied molecular orbitals (HOMOs) were calculated using density functional theory (DFT) methods with the B3LYP exchange-correlation functional and the 6-31G(d) basis set using the procedure embedded in Wavefunction (Irvine, CA, USA) Spartan 14. During the calculation, the intramolecular hydrogen bond (IHB) provided the most stable conformation for each molecule.

## 3. Results and Discussion

### 3.1. Chemistry

**Figure 2:**
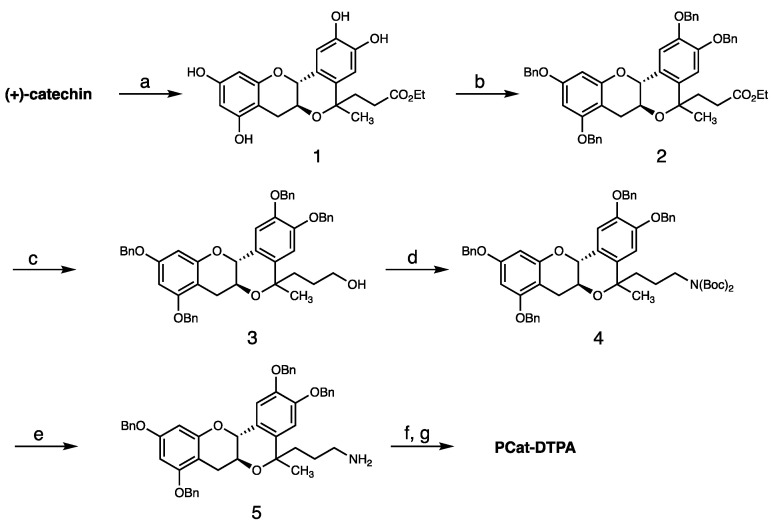
Synthesis of PCat-DTPA. Reagents and conditions: (a) 4-oxovalerate, TMSOTf, THF, −20 °C; (b) benzyl bromide, K_2_CO_3_, DMF, rt; (c) LiAlH_4_, THF, rt; (d) di-*tert*-butyliminodicarboxylate, Ph_3_P, DEAD, toluene, 0 °C; (e) TFA, rt; (f) DTPA, DMT-MM, DMSO, rt; (g) 20% Pd(OH)_2_, H_2_, MeOH, rt.

The planar catechin moiety (1) in PCat-DTPA, in which the steric structure of catechin is fixed to a plane, was obtained by the oxa-Pictet–Spengler reaction between catechin and ethyl revulinate (Figure 2). That is, catechin and ethyl revulinate in THF was treated with TMSOTf to form compound **1**. After protecting the hydroxyl group of compound **1** with a benzyl group using benzyl bromide, the ester structure was reduced to the primary alcohol form (3) by LiAlH_4_. Next, compound **3** was subjected to the Mitsunobu reaction using (Boc)_2_NH as pronucleophile in the presence of Ph_3_P and DEAD; the hydroxyl group was converted to an amino group by further treatment with TFA. Subsequently, the condensation reaction between the amino group of compound **5** and the carboxyl group of DTPA proceeded rapidly using the condensing agent DMT-MM, followed by a deprotection reaction by contact hydrogen reduction with catalytic amount of 20% Pd(OH)_2_ to synthesize PCat-DTPA.

### 3.2. Radical Scavenging Activity of PCat-DTPA toward DPPH^•^

**Figure 3:**
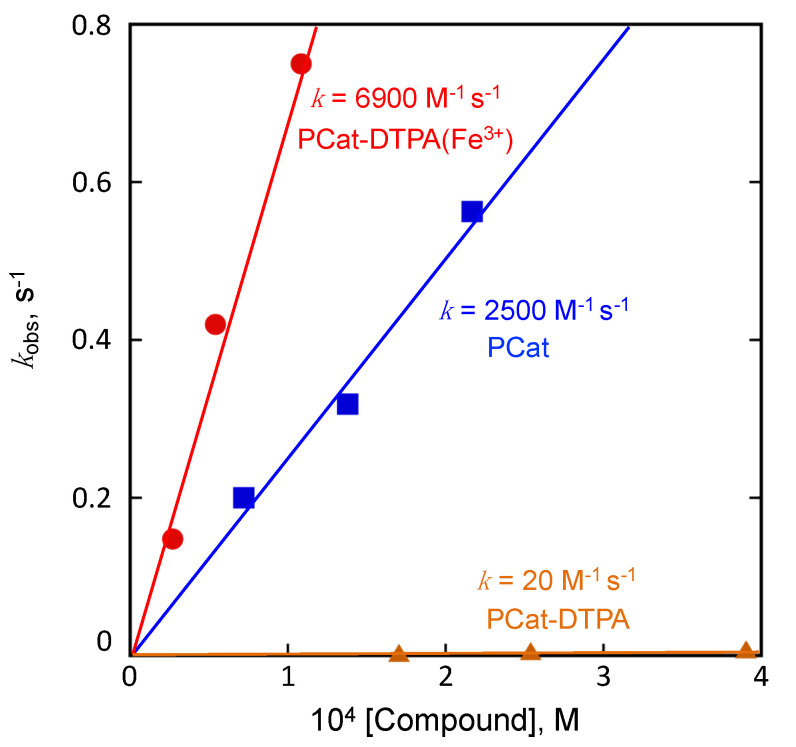
Plot of the pseudo-first-order rate constant (*k*_obs_) vs. the concentration of PCat (blue), PCat-DTPA (orange), and PCat-DTPA(Fe^3+^) (red) for the radical scavenging reaction toward DPPH^•^.

The anti-radical efficiency of PCat-DTPA and related compounds was evaluated by performing a radical scavenging reaction against the DPPH^•^, a model radical for ROS. Under pseudo-first-order reaction conditions using a compound at a concentration 10 times higher than that of the DPPH^•^, the pseudo-first-order rate constant (*k*_obs_) for the DPPH radical was determined by measuring the decrease in absorption at 517 nm. As the pseudo-first-order rate constant increased in proportion to the compound’s concentration, the compound’s reaction rate was determined from the slope of the rate constant (Figure 3). Therefore, the *k* of PCat was 2500 M^−1^s^−1^ and that of PCat-DTPA was 20 M^−1^s^−1^, indicating that the binding of DTPA significantly weakens the radical scavenging activity of PCat. However, when an equal amount of Fe^3+^ was added to PCat-DTPA before the reaction with DPPH, the *k* of PCat-DTPA was 6900 M^−1^s^−1^, which was 2.7 times stronger than that of PCat. This indicates that PCat-DTPA, which shows little radical scavenging activity due to DTPA, turns on the radical scavenging reaction by Fe^3+^ coordination to DTPA; the radical scavenging activity was notably stronger than that of PCat. Therefore, we proposed that Fe^3+^ coordinates with PCat-DTPA to enhance the radical scavenging activity of PCat and remove the inhibitory effect of DTPA on the radical scavenging activity of PCat. However, neither DTPA nor Fe^3+^-coordinated DTPA showed radical scavenging activity against DTPA (data not shown). Therefore, the potent radical scavenging activity of PCat-DTPA by Fe^3+^ coordination may not be due to the additional radical scavenging activity of Fe^3+^-coordinated DTPA but rather the acceleration of the radical scavenging reaction of PCat by the bound Fe^3+^-coordinated DTPA.

### 3.3. Antioxidant Activity of PCat-DTPA toward Free Radicals Generated from the Fe-XO/HX System

**Figure 4:**
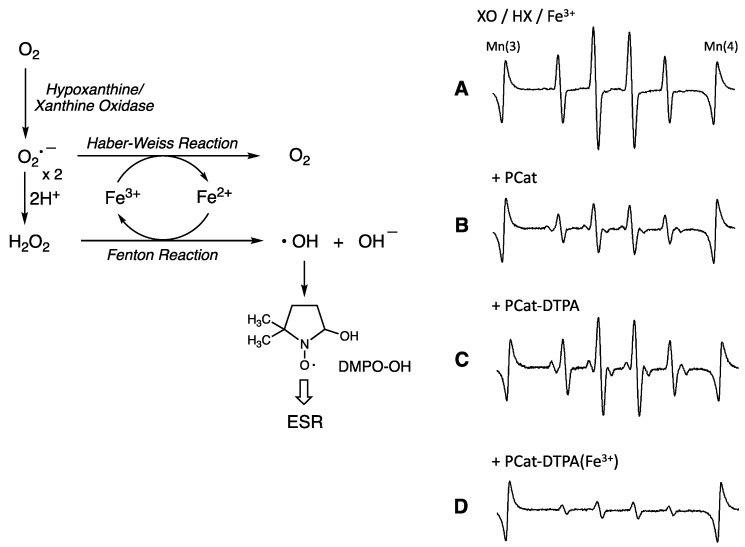
Effects of PCat analogs on hydroxy radical adduct formation of DMPO in the Fe-XO/HX system. ESR spectra of spin adducts were observed during the reaction of hypoxanthine (0.2 mM), xanthine oxidase (0.05 U/mL), and FeCl_3_ (0.01 mM) in the absence (**A**) or presence of PCat analogs (0.1 mM; (**B**): PCat, (**C**): PCat-DTPA, (**D**): PCat-DTPA(Fe^3+^)).

For PCat-DTPA to exert a preventive effect against the onset and progression of oxidative stress-related diseases, it is important that PCat-DTPA removes iron ions from the ROS-generating system in vivo and exhibits potent antioxidant activity against the iron-mediated ROS-generating system in vivo after coordination with iron ions. Therefore, we investigated the antioxidant capacity of Fe^3+^-coordinated PCat-DTPA against ROS generated from the iron-containing XO and HX (Fe-XO/HX), a model system of ROS generation in vivo. XO is an enzyme that catalyzes the oxidation of HX to xanthine and xanthine to uric acid, a process that reduces molecular oxygen and produces O_2_^•−^; the generated superoxide spontaneously forms H_2_O_2_ via dismutation. Furthermore, the generated superoxide interacts with H_2_O_2_ in the presence of Fe^3+^ to form the most potent cytotoxic hydroxyl radical (^•^OH) via the Haber–Weiss (Fenton) reaction [33]. By adding DMPO as a spin-trapping agent, the hydroxyl radicals formed in the Fe-XO/HX system can be detected by the ESR signal of 1:2:2:1 quartet with hyperfine coupling constants (*a*_N_ = *a*_H_ = 14.9G), indicative of the scavenging of hydroxyl radicals by the DMPO-OH spin adduct. When PCat was added to the Fe-XO/HX system, a decrease in the ESR signal for DMPO-OH was observed (Figure 4). Because PCat does not chelate with iron (data not shown), the decreased ESR signal is responsible for the scavenging effect of PCat on the hydroxyl radicals generated in the Fe-XO/HP system. In the presence of PCat conjugated with DTPA (PCat-DTPA), the ESR signal intensity of DMPO-OH in the Fe-XO/HX system was only slightly reduced, indicating a minor antioxidant effect. As Fe^3+^ in the Fe-XO/HX system is coordinated with DTPA, PCat-DTPA cannot remove Fe^3+^ to generate hydroxyl radicals. Therefore, it is believed that PCat-DTPA with weak antioxidant activity could not scavenge the hydroxyl radicals generated in the Fe-XO/HX system. Consequently, the ESR signal for DMPO-OH was not considerably reduced. In contrast, PCat-DTPA coordinated with Fe^3+^ reduced the signal intensity of DMPO-OH generated in the Fe-XO/HX system to an even greater extent than PCat, exhibiting highly potent antioxidant activity, similar to the results of radical scavenging activity against DPPH. These results indicate that the difference in the antioxidant activity of PCat, PCat-DTPA, and its Fe^3+^-coordinated activity is almost consistent with the radical scavenging activity against DPPH radicals in the Fe-XO/HX system. This finding also implies that these compounds do not affect the mechanism of hydroxyl radical generation in the Fe-XO/HX system but directly scavenge the hydroxyl radicals generated in this system.

### 3.4. Effect of PCat-DTPA on Oxidative Damage of pBR322DNA by Fenton Reaction

**Figure 5:**
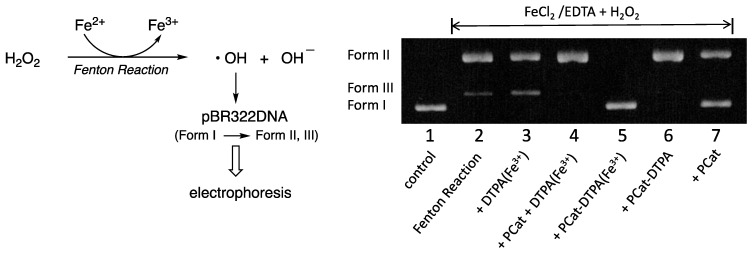
Effects of PCat analogs on pBR322 DNA strand breaks induced by the Fenton reaction system. DNA subjected to the Fenton reaction in the absence (lanes 1 and 2) or presence of PCat analogs (lanes 3–7) was incubated at 37 °C for 0.5 h with 10 µM FeCl_3_, 10 µM EDTA, and 10 mM H_2_O_2_ in the absence or presence of 100 µM of the test compounds.

ROS generated through the Fenton and Haber–Weiss reactions play a significant role in oxidative stress, causing numerous degenerative diseases, such as cancer, diabetes, Parkinson’s, and Alzheimer’s [34,35,36]. The highly reactive hydroxyl radicals generated by iron-mediated Fenton reactions damage numerous molecules in cells and play a critical role in inducing DNA oxidative damage. During oxidative damage of pBR322DNA by hydroxyl radicals, induction of single-strand breaks in the supercoiled double-stranded DNA leads to the formation of open circular DNA, while forming a linear form of DNA indicates double-strand breaks, suggesting further damage to the open circular DNA. Thus, the Fenton reaction involving Fe^2+^ and H_2_O_2_ for the generation of hydroxyl radicals was used to investigate whether PCat-DTPA and its Fe-coordinated forms could protect against DNA damage. For the Fenton reaction to function effectively, EDTA was employed for chelation with Fe^2+^, preventing the repression of hydroxyl radical generation due to the coordination of PCat-DTPA to Fe^2+^ for the Fenton reaction. Incubating DNA with H_2_O_2_ and Fe^2+^/EDTA converted all supercoiled DNA into open circular forms, indicating that DNA breaks can be induced by the H_2_O_2_/Fe/EDTA system (Figure 5). Although PCat exhibited scavenging activity toward DPPH and hydroxyl radicals generated in the Fe-XO/HP system, adding PCat in the Fenton system did not significantly inhibit the conversion of supercoiled DNA to the open-circle form. In contrast, PCat-DTPA showed very low radical scavenging activity against DPPH and hydroxyl radicals in the Fe-XO/HX system; however, PCat-DTPA inhibited the conversion of supercoiled DNA to the open circular form by approximately half of the DNA cleavage reactions by the Fenton system. Furthermore, the coordination of Fe^3+^ to PCat-DTPA resulted in the complete inhibition of the conversion of supercoiled DNA to an open circular form, indicating that PCat-DTPA is transformed into a potent antioxidant by coordinating Fe^3+^ against DPPH and hydroxyl radicals in the Fe-XO/HX system. Fe^3+^-coordinated DTPA further accelerated DNA damage in the Fenton system, as a significant formation of an open circular form of DNA (with a slight generation of a linear form) was observed upon its addition to the Fenton system. Furthermore, the coexistence of PCat and Fe^3+^-coordinated DTPA in the Fenton system did not inhibit the DNA damage reaction, similar to PCat-DTPA(Fe^3+^), as only an open circular form of DNA was observed. These results suggest that the potent antioxidant activity of PCat-DTPA(Fe^3+^) can be attributed to the enhancement of the radical scavenging activity of PCat by intramolecular DTPA(Fe^3+^).

### 3.5. DFT Calculations for PCat-DTPA

**Figure 6:**
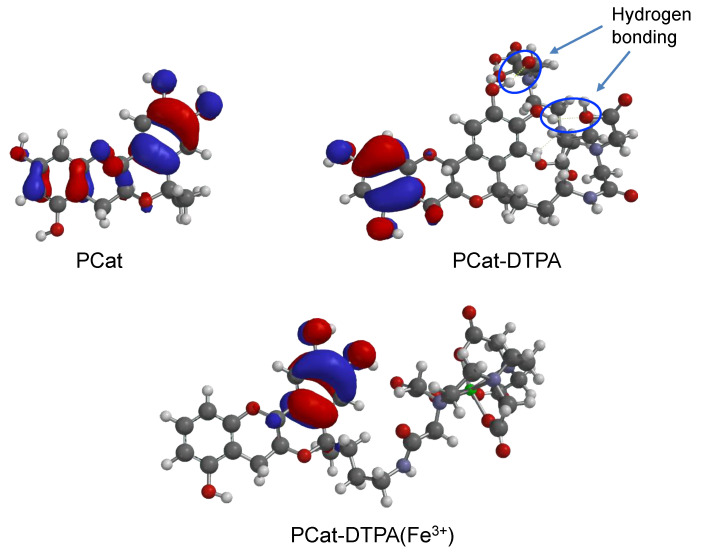
The optimized geometries and HOMOs of PCat, PCat-DTPA, and PCat-DTPA(Fe^3+^) using the density functional theory at the B3LYP/6-31G*.

Since the antioxidant action of the phenolic antioxidants catechin and quercetin is due to ROS reduction by catechol, the orbital composition of their HOMOs may affect their antioxidant activities. Moreover, the charge density of catechin is primarily localized on the aromatic ring A, with less involvement of the chromane moiety [37]. However, the electron density in PCat with more potent radical scavenging activities than catechin is distributed in all rings, with a moderate increase in the catechol structure, owing to the nearly planar arrangement. Therefore, density theory calculations were performed to reveal the HOMOs of PCat-DTPA and its Fe^3+^ coordinate, including their equilibrium geometries (Figure 6). The most stable structure of PCat-DTPA, optimized at the DFT theoretical level, formed an IHB between the hydroxyl hydrogen in catechol and the carboxy oxygen in DTPA. The charge density on the HOMO level in PCat-DTPA was distributed in the A ring and not in the catechol structure; however, in PCat, it was distributed over the entire molecule. Therefore, the weaker antioxidant activity of PCat-DTPA compared to PCat may be due to the acidic functional group in DTPA, the carboxy group, which localizes the charge density distribution of the HOMO to the A ring by forming IHBs with catechol. In contrast, in PCat-DTPA with Fe^3+^ coordination, the IHB formed in PCat-DTPA was no longer present, and the charge density on the HOMO was localized on the catechol ring. Therefore, the catechol structure of PCat-DTPA(Fe^3+^) with increased charge density could readily reduce ROS. The stronger antioxidant effect of PCat-DTPA(Fe^3+^) than PCat is probably due to the higher charge density distribution of the HOMO of the catechol structure in PCat-DTPA(Fe^3+^). There are two possible mechanisms for the scavenging of ROS by PCat-DTPA(Fe^3+^). One is the direct reduction of ROS by the catechol with increased electron density, and the other is the electron transfer from catechol to ROS mediated by Fe^3+^ coordinated to DTPA. The detailed antioxidant mechanism of this compound is currently being investigated.

## 4. Conclusions

Phenolic antioxidants, such as catechins, exhibit antioxidant activity by scavenging and reducing ROS generated in the human body; however, they have not been sufficiently effective in preventing or treating diseases associated with oxidative stress. To effectively suppress ROS toxicity, it is necessary to directly scavenge ROS generated in the body and regulate the mechanism of ROS generation. Diseases related to oxidative stress often show accumulation of metals, such as iron, at the site of disease onset; the metal acts as a catalyst to generate ROS by reducing oxygen molecules. Therefore, if metals can be removed from the pathological site of oxidative stress and the generated ROS can be strongly scavenged, oxidative stress-related diseases can be prevented and treated. We designed and synthesized PCat-DTPA in which the metal chelator DTPA was combined with a planar catechin, enhancing the antioxidant activity of natural catechins by immobilizing its steric structure to be planar. As the HOMO was localized on the A ring due to IHB formation between the catechol structure and DTPA, PCat-DTPA showed almost no antioxidant activity. However, when the coordination of Fe^3+^ broke the hydrogen bond to DTPA, the HOMO was localized on the catechol structure, resulting in PCat-DTPA(Fe^3+^) showing stronger radical scavenging activity than PCat. PCat-DTPA does not normally exhibit antioxidant activity; however, at the site of pathogenesis involving Fe^3+^, DTPA bound to PCat could remove Fe^3+^ by coordination, inhibiting ROS generation through the Fenton and Haber–Weiss reactions. In addition, PCat-DTPA could exert a strong radical scavenging effect triggered by coordination with Fe^3+^, showing excellent antioxidant activity against ROS-generating pathologies. In general, phenolic antioxidants with strong antioxidant activity can be made to exert pro-oxidant effects, which may be related to the toxic manifestation of antioxidant activity. PCat-DTPA does not exhibit antioxidant activity when the metal is not coordinated, suggesting that it is a safe antioxidant that does not have pro-oxidant effects in normal tissues. On the other hand, in pathological sites where metals are involved in the onset of oxidative stress, metal coordination triggers a strong antioxidant effect, suggesting that PCat-DTPA may selectively exert a potent therapeutic effect son oxidative stress-induced lesions in disease. Finally, in the pathogenesis of Fe^3+^-related oxidative stress, PCat-DTPA is expected to be useful as a chemical scaffold or lead compound for the prevention and treatment of these diseases because of its ability to remove Fe^3+^ and its strong radical scavenging activity triggered by Fe^3+^ coordination. In order to develop PCat-DTPA as a therapeutic agent, modifications that consider the pharmacokinetics and cell membrane permeability, such as ester prodrugs to increase lipid solubility, are necessary. Research is ongoing to elucidate the details of the radical scavenging mechanism of PCat-DTPA and develop more effective new compounds for clinical applications.

## Data Availability

All data are contained in the article.
